# SUBJECTIVELY AND OBJECTIVELY ASSESSED SLEEP IN OLDER ADULTS WITH AND WITHOUT MILD COGNITIVE IMPAIRMENT

**DOI:** 10.1093/geroni/igae098.3527

**Published:** 2024-12-31

**Authors:** Kworweinski Lafontant, James Brightman, Jethro Raphael Suarez, Amber Blount, David Fukuda, Jeffrey Stout, Ladda Thiamwong

**Affiliations:** University of Central Florida, Orlando, Florida, United States; University of Central Florida, Orlando, Florida, United States; University of Central Florida; University of Central Florida, Orlando, Florida, United States; University of Central Florida, Orlando, Florida, United States; University of Central Florida, Orlando, Florida, United States; University of Central Florida, Orlando, Florida, United States

## Abstract

Mild cognitive impairment (MCI) has been proposed as a potential confounding factor when subjectively assessing sleep in older adults. However, it is unclear whether older adults with MCI are less accurate in self-reporting sleep than those without MCI. This cross-sectional investigation compared the mean absolute difference (MAD) between subjectively and objectively assessed sleep duration, efficiency, and latency among community-dwelling low-income older adults with MCI (n = 13, females = 11, age = 77.5 ± 7.83 years, BMI = 29.0 ± 4.29 kg/m2) and those without MCI (n = 77, females = 69, age = 73.8 ± 7.07 years, BMI = 30.6 ± 7.38 kg/m2). The Pittsburgh Sleep Quality Index (PSQI), wrist-worn actigraphy (GT9X Link, ActiGraph LLC, Pensacola, FL, USA) and the Rowland Universal Dementia Assessment Scale were used to assess subjective sleep, objective sleep, and MCI, respectively. A Kolmogorov-Smirnov test revealed that data were non-normally distributed, so Mann-Whitney U tests were used for between-group comparisons. There was a significant difference in sleep duration MAD between older adults with MCI (6.04 ± 2.22 hrs) and those without MCI (6.42 ± 1.47 hrs; p = 0.03). However, sleep efficiency and latency MAD were not significantly different between the groups. While older adults with MCI may be less accurate at recalling sleep duration, their ability to distinguish sleep duration from time spent in bed is comparable to that of older adults without MCI. Future research should include older adults with MCI when assessing specific subdomains of sleep quality subjectively.

